# Neoadjuvant FOLFOX chemotherapy combined with radiotherapy followed by radical resection in patients with locally advanced colon cancer

**DOI:** 10.1186/s13014-017-0790-3

**Published:** 2017-03-07

**Authors:** Chun-Ming Huang, Ming-Yii Huang, Cheng-Jen Ma, Yung –Sung Yeh, Hsiang-Lin Tsai, Ching-Wen Huang, Chih-Jen Huang, Jaw-Yuan Wang

**Affiliations:** 1 Department of Radiation Oncology, Kaohsiung Medical University Hospital, Kaohsiung Medical University, Kaohsiung, Taiwan; 2 Graduate Institute of Medicine, College of Medicine, Kaohsiung Medical University, Kaohsiung, Taiwan; 3 Department of Radiation Oncology, Faculty of Medicine, College of Medicine, Kaohsiung Medical University, Kaohsiung, Taiwan; 4 Department of Surgery, Division of General and Digestive Surgery, Kaohsiung Medical University Hospital, Kaohsiung Medical University, Kaohsiung, Taiwan; 5 Department of Surgery, Division of Colorectal Surgery, Kaohsiung Medical University Hospital, Kaohsiung Medical University, No. 100 Tzyou 1st Road, Kaohsiung, 807 Taiwan; 6 Graduate Institute of Clinical Medicine, College of Medicine, Kaohsiung Medical University, Kaohsiung, Taiwan; 7 Department of Surgery, Division of Trauma and Critical Care, Kaohsiung Medical University Hospital, Kaohsiung Medical University, Kaohsiung, Taiwan; 8 Department of Emergency Medicine, Kaohsiung Medical University Hospital, Kaohsiung Medical University, Kaohsiung, Taiwan; 9 Department of Surgery, Faculty of Medicine, College of Medicine, Kaohsiung Medical University, Kaohsiung, Taiwan; 10 Center for Biomarkers and Biotech Drugs, Kaohsiung Medical University, Kaohsiung, Taiwan; 11 Research Center for Environmental Medicine, Kaohsiung Medical University, Kaohsiung, Taiwan; 12 Research Center for Natural Products & Drug Development, Kaohsiung Medical University, Kaohsiung, Taiwan

**Keywords:** Colon cancer, Oxaliplatin, Chemoradiotherapy, Pathologic complete response

## Abstract

**Background:**

Patients with locally advanced colon cancer (LACC) have a relatively poor prognosis despite radical resection and adjuvant chemotherapy. This study investigated the treatment efficacy and toxicity of neoadjuvant chemoradiotherapy in patients with LACC.

**Methods:**

We retrospectively reviewed 36 patients with LACC preoperatively treated with chemotherapy and radiotherapy. Patients were administered chemoradiotherapy, which comprised radiotherapy and neoadjuvant chemotherapy involving a 5-fluorouracil, leucovorin, and oxaliplatin regimen every 2 weeks.

**Results:**

Median age was 64 years (45–86 years) and median follow-up period was 23.5 months (5.0–49.1 months). Seven (19.4%) patients developed grade 3 or 4 adverse events during neoadjuvant concurrent chemoradiotherapy. Pathologic responses were not evaluated in two patients who did not undergo radical resection. Of the 34 patients who underwent surgery, nine (26.4%) achieved a pathologic complete response (pCR). The 2-year estimated overall survival and disease-free survival rates were 88.7% and 73.6%, respectively.

**Conclusions:**

Our results demonstrated that neoadjuvant chemoradiotherapy is feasible and safe. A prominent pCR rate with an acceptable toxicity profile suggests that the multimodality therapy might be a treatment option for patients with LACC.

## Background

Worldwide, colorectal cancer is the third most commonly diagnosed cancer in men and the second most commonly diagnosed cancer in women [1]. In Taiwan, colorectal cancer is the most common cancer, with a rapid increase in its prevalence, and is the third leading cause of cancer-related death [2]. Complete tumor removal surgery with a margin negative resection (R0) is the only curative modality for localized colon cancer. However, treatment results for locally advanced colon cancer (LACC), which is clinically defined as a primary tumor that directly invades adjacent structures or by the presence of extensive nodal involvement that renders curative resection infeasible, remain disappointing despite recent developments in surgery with subsequent adjuvant chemotherapy [3, 4]. The 5-year survival rates for patients with stage IIC, IIIB, and IIIC LACC were reported to be 37.3%, 46.3 and 28%, respectively [5], which has prompted researchers to investigate new treatment approaches for LACC in order to resolve the problems of markedly low survival rates resulting from tumor invasion to adjacent organs or extensive lymph node metastasis.

Neoadjuvant concurrent chemoradiotherapy (CCRT) instead of initial surgery is the current standard treatment for locally advanced rectal cancer, which has been well-established by randomized trials [6, 7]. The Chinese FOWARC randomized phase III trial demonstrated that patients with locally advanced rectal cancer treated with neoadjuvant 5-fluorouracil, leucovorin, and oxaliplatin (FOLFOX)-based CCRT achieved a higher pathologic complete response (pCR) rate than those treated with fluorouracil-based CCRT or perioperative FOLFOX alone [8]. However, the role of radiotherapy in the treatment of LACC remains unclear. Although benefits of postoperative radiotherapy have been reported in selected groups of patients with LACC [9, 10], the intergroup 0130 trial demonstrated that there was no difference in overall survival and disease-free survival between LACC patients receiving postoperative chemotherapy alone and those receiving postoperative CCRT [11]. In addition, hematologic toxicity was higher in the CCRT group. However, the intergroup 0130 trial has been criticized for its slow accrual and a large number of ineligible patients. For neoadjuvant CCRT in patients with LACC invading adjacent organs or extensive lymph node metastasis, the advantages of neoadjuvant CCRT have been reported in limited case reports and two small case series [12–15]. On the basis of our previous reports, which indicated that neoadjuvant FOLFOX-based CCRT resulted in a pCR rate of up to 31.6% in patients with locally advanced rectal cancer [16], we adopted the same treatment modality in patients with LACC to potentially improve their oncologic outcomes.

The present study investigated the treatment efficacy, toxicity and short-term oncologic outcome of neoadjuvant FOLFOX-based CCRT in patients with LACC.

## Methods

The present study included 36 consecutive patients who received a histopathological diagnosed of colon adenocarcinoma and were treated with neoadjuvant CCRT followed by radical resection with curative intent in a single institute between January 2012 and June 2016. Multidisciplinary cancer conferences have recommended that patients with potentially suitable for incomplete resection of LACC should receive neoadjuvant CCRT. The potential for incomplete resection was defined by a T3 tumor with extramural extension of >5 mm or a T4 tumor diagnosed by imaging studies. Other inclusion criteria were colon cancer located above 15 cm from the anal verge, an Eastern Cooperative Oncology Group score of 0–2, and no evidence of distant metastasis at diagnosis. The exclusion criteria were a history of previous or synchronous malignancies other than nonmelanoma skin cancer and the presence of serious medical comorbidities that may influence treatment compliance. Medical records were reviewed to analyze the treatment efficacy, toxicity and short-term oncologic outcomes. The present study was approved by the Institutional Ethics Committee of our hospital. Pretreatment evaluation entailed a complete medical history review and physical examination, colonoscopy, tumor biopsy, chest radiography, abdominal and pelvic computed tomography (CT) with or without magnetic resonance imaging, carcinoembryonic antigen (CEA) level assessment, and routine laboratory tests.

### Preoperative treatment

The concurrent chemotherapy regimen was a biweekly schedule of FOLFOX. Each cycle of FOLFOX consisted of oxaliplatin (85 mg/m^2^) and folinic acid (400 mg/m^2^) infusion on day 1 followed by a 46-h infusion of 5-fluorouracil (5-FU, 2800 mg/m^2^) repeated every 2 weeks. All patients received concurrent chemotherapy and radiotherapy. After completion of radiotherapy, all patients received chemotherapy twice weekly until surgery. Patients underwent surgery about 4 weeks after completing preoperative chemotherapy.

All patients underwent a planning CT in the supine position and were immobilized with custom thermoplastic immobilization devices before initiating radiotherapy. Target volumes were delineated according to the International Commission on Radiation Units and Measurements reports 50 and 62 [17]. The gross tumor volume (GTV) was defined as the macroscopic tumor and enlarged lymph nodes visible on diagnostic CT images. The clinical target volume (CTV) was the GTV plus a 15- to 20-mm margin, and the planning target volume was the CTV plus a 10- to 15-mm margin. Organs at risk (OAR), namely kidney, small bowel, liver, and spinal cord, were contoured. A radiation dose of 45–50.4 Gy was administered in 25–28 fractions. The dose constraints for OARs were as follows: the V30 of the liver was kept at <30%; the mean dose and V20 of the kidneys were restricted to <15 Gy and <30%, respectively; the volume of small bowel receiving >50 Gy was limited to <1 cc; and the maximal dose to the spinal cord was restricted to <45 Gy.

### Surgery and pathology review

Patients underwent elective surgery at >6 weeks after completion of radiotherapy. The pathologic tumor (T) and nodal (N) stages (ypT and ypN, respectively) of the tumor, histological grade, lymphovascular invasion, perineural invasion, tumor regression grade (TRG), and status of the circumferential, proximal, and distal resection margins were documented. The tumor response after CCRT was assessed according to the American Joint Committee on Cancer (AJCC) system as follows [18]: Grade 0, no residual cancer cells; Grade 1, single cell or small group of cancer cells (major regression); Grade 2, residual cancer with desmoplastic response (moderate regression); and Grade 3, minimal evidence of tumor response. A circumferential resection margin (CRM) of <1 mm was defined as an involved CRM [19]. A pCR was defined as the absence of viable cancer cells in the pathological specimens, including primary tumor and lymph nodes (ypT0N0), after neoadjuvant CCRT.

### Postoperative chemotherapy

Fifteen patients received an adjuvant chemotherapy regimen of FOLFOX. Adjuvant UFUR (tegafur and uracil) and capecitabine were administered in 11 and 5 patients, respectively; 3 patients did not receive postoperative chemotherapy. Two patients were transferred to the folinic acid, 5-FU, and irinotecan (FOLFIRI) regimen after laparotomy because of a poor response to the FOLFOX-based CCRT and the development of distant metastasis.

### Toxicity evaluation and follow-up

During CCRT and in postoperative follow-up, acute adverse events at each visit were graded according to the Common Terminology Criteria for Adverse Events (CTCAE), version 3.0 (http://ctep.cancer.gov/reporting/ctc.html). Late radiation toxicity was scored using the Radiation Therapy Oncology Group Late Radiation Morbidity Scoring System. After surgery, patients were followed monthly for 6 months and subsequently once every 2–3 months to date.

### Endpoints and statistics

The pCR rate was the primary endpoint of the study. Secondary endpoints were multimodality therapy-associated toxicities, TGR, and R0 resection rate. Downstaging was determined according to the response between the clinical T or N stage before neoadjuvant CCRT and the postoperative pathological T or N stage.

Descriptive statistics are presented as proportions and medians. The chi-squared test and Fisher’s exact test were used to compare the categorical data, and normally distributed continuous variables were analyzed using the Student *t* test. Disease-free survival (DFS) was measured from the date of onset of CCRT to the date of any type of recurrence or last follow-up. Overall survival (OS) was defined as the time from the onset of CCRT until death due to any cause or until the final follow-up. Survival rates were estimated using the Kaplan–Meier method. Data analyses were performed using JMP software (version 9.0, SAS Institute Inc., Cary, NC, USA).

## Results

The median age was 64 years (45–86 years). Most tumor locations were the sigmoid colon (58.4%) followed by the ascending colon. Four patients received three-dimensional conformal radiotherapy using either three or four fields. Volumetric-modulated arc therapy (VMAT) and helical tomotherapy were administered to 22 and 10 patients, respectively. Table 1 lists the patient and treatment characteristics.

**Table 1:** Patient and treatment characteristics (*N* = 36)

Age, median (years, range)	64 (45–86)
Gender	
Male	20 (55.6)
Female	16 (44.4)
Location	
Sigmoid colon	21 (58.4)
Ascending colon	12 (33.3)
Transverse colon	3 (8.3)
Clinical tumor depth	
T3	13 (36.1)
T4a	13 (36.1)
T4b	10 (27.8)
Clinical lymph node metastasis	
N0	1 (2.7)
N1	16 (44.5)
N2	19 (52.8)
UICC staging	
IIC	1 (2.7)
IIIB	15 (41.7)
IIIC	20 (55.6)
Pretreatment CEA (ng/mL)	
≦ 5	19 (52.8)
> 5	17 (47.2)
Ileosotmy/colostomy prior to therapy	
Yes	19 (52.7)
No	17 (47.3)
Radiotherapy (dose/fractions)	
45 Gy/25	6 (16.7)
50 Gy/25	25 (69.5)
50.4Gy/28	4 (11.1)
46.8Gy/26	1 (2.7)
Post-operative chemotherapy	
FOLFOX	15 (41.7)
UFUR	11 (30.6)
Capecitabine	5 (13.9)
FOLFIRI	2 (5.4)
None	3 (8.4)
BMI kg/m^2^ (median) (range)	22.4 (14.8–41.8)
RT-Surgery interval week (median) (range)	11 (6–25)

Data are presented as n (%), unless otherwise indicated

*CEA* carcinoembryonic antigen, *FOLFOX* fluorouracil, leucovorin, and oxaliplatin, *UFUR* Tegafur and Uracil, *FOLFIRI* fluorouracil, leucovorin, and irinotecan, *UICC* Union for International Cancer Control, *BMI* body mass index, *RT* radiotherapy

### Acute toxicity and treatment compliance

Table 2 presents the acute adverse events during neoadjuvant CCRT. Overall, seven (19.4%) of the 36 patients experienced grade 3 or 4 adverse events during neoadjuvant CCRT. Leukopenia was defined according to the CTCAE, version 3.0, and leucopenia (83.2%) was the most common adverse event; however, most events (69.4%) were of grade 1 or 2 and were manageable in all patients. For non-hematologic toxicity, fatigue and nausea were the leading side effects caused by CCRT. Moreover, there were no grade 4 adverse events or neoadjuvant CCRT-related deaths.

**Table 2:** Toxicities during neoadjuvant treatment (*N* = 36)

Toxicity	Grade 1		Grade 2		Grade 3	
	No.	%	No.	%	No.	%
All	28	77.8	30	83.3	7	19.4
Fatigue	22	61.1	0	0.0	0	0.0
Hematologic						
Hemoglobin	11	30.5	10	27.7	6	16.7
Leukocytes	6	16.7	19	52.7	5	13.8
Gastrointestinal						
Nausea	14	38.8	4	11.1	1	2.7
Vomiting	3	8.3	5	13.8	0	0.0
Diarrhea	11	30.5	6	16.7	1	2.7
Paresthesia	10	27.7	7	19.4	0	0.0
Oral mucositis	6	16.7	2	5.5	0	0.0
Dermatitis	8	22.2	2	5.5	0	0.0

Of all the patients, only one did not complete the prescribed radiation course (50.4 Gy); radiotherapy was interrupted at 46.8 Gy with two fractions remaining because of grade 3 diarrhea and grade 2 vomiting. The patient with sigmoid colon cancer did not complete the radiotherapy during the study period; however, he finally underwent surgery 51 days after completion of radiotherapy. The median radiotherapy duration was 35 days (32–49 days). Two patients temporarily discontinued preoperative chemotherapy because of neutropenic fever (n = 1) and grade 3 diarrhea with dehydration (*n* = 1), and both of the two patients continued scheduled preoperative chemotherapy after management of those adverse events.

### Surgery and pathologic response

The median interval between radiotherapy completion and surgery was 11 weeks (6–25 weeks). All but two patients underwent surgery after neoadjuvant CCRT. Of the 34 patients receiving surgery, sigmoidectomy was performed in 17 (50%), right hemicolectomy in 12 (35.3%), and left hemicolectomy in five (14.7%) patients. Multivisceral resection was performed in 6 patients: 2 patients underwent colectomy plus partial cystectomy; 2 patients underwent colectomy plus resection of small intestine; 1 patient underwent colectomy plus partial gastrectomy; 1 patient underwent colectomy plus extensive resection of visceral peritoneum. Of the 4 patients diagnosed with bladder invasion, 2 received a partial cystectomy and 2 underwent a simple colectomy while preserve the bladder intact. Two patients did not undergo radical resection after neoadjuvant therapy. One patient with sigmoid colon cancer accompanied by uterus and left ureter invasion did not undergo radical resection after neoadjuvant treatment because of tumor invasion to the common iliac artery, which was observed during the surgery. Therefore, the tumor was not resected and the patient was administered 10 cycles of FOLFOX. Thereafter, FOLFIRI was administered as a second-line chemotherapy, and the patient’s condition was stable for 15 months at the final follow-up. The other patient with ascending colon cancer, T4aN2bM0, developed peritoneal carcinomatosis, which was revealed by CT images, after completion of the neoadjuvant FOLFOX-based CCRT; therefore, surgery was not performed. Two cycles of the FOLFIRI regimen were administered to the patients for disease control; however, the patient died because of tumor progression at 7 months after diagnosis.

Table 3 lists the pathologic results and treatment efficacy. Final pathologic analysis revealed that of the 34 patients, nine (26.4%) achieved ypT0 (no viable tumor in the primary site) and 28 (82.4%) achieved ypN0. The median number of lymph nodes retrieved was 11 (3–26). The resection margin was free of cancer cells in 31 (91.2%) of the 34 patients. Using a univariate analysis of the correlation between positive surgical margins and clinicopathologic features, the unfavorable tumor regression (TRG 3–4 versus TRG 0–1; *p* = 0.030), CEA level > 5 (*p* = 0.041), and ypT4 disease (*p* = 0.042) were associated with positive surgical margins. Of the 34 patients who underwent surgery, pCR, major regression (TRG 1), and moderate regression (TRG 2) were achieved in 9 (26.4%), 11 (32.4%), and 7 patients (20.6%), respectively, and 14 patients (41.2%) downstaged to the ypT0-2 stage. Comparison between the changes in the clinical and pathologic stages revealed that TN downstaging was achieved in 29 (85.3%) of the 34 patients; primary tumor and nodal downstaging were achieved in 24 (70.6%) and 31 (91.2%) of the 34 patients, respectively.

**Table 3:** Pathological results and tumor response to neoadjuvant treatment (*N* = 34)^b^

	No. (%)
ypT	
0	9 (26.4)
1	0 (0)
2	5 (14.7)
3	17 (50.0)
4	3 (8.9)
ypN	
0	28 (82.4)
1	5 (14.7)
2	1 (2.9)
Median number of resected nodes^a^	11 (3–26)
Median number of involved nodes^a^	0 (0–6)
Lymph node with extranodal involvement	4 (11.7)
Lymphovascular invasion	
Yes	5 (14.7)
No	29 (85.3)
Perineural invasion	
Yes	2 (5.9)
No	32 (94.1)
Tumor differentiation	
Well	2 (5.9)
Moderately	28 (82.4)
Poorly	4 (11.7)
Resection margin (CRM)	
Negative	31 (91.2)
Positive	3 (8.8)
Pathologic complete response	
Yes	9 (26.4)
No	25 (73.6)
Tumor regression grade	
0	9 (26.4)
1	11 (32.4)
2	7 (20.6)
3	7 (20.6)
ypT0-2 vs. ypT3-4	
ypT0-2	14 (41.2)
ypT3-4	20 (58.8)
Pathologic T stage	
Downstaging	24 (70.6)
Stable	10 (29.4)
Progressive	0 (0)
Pathologic N stage	
Downstaging	31 (91.2)
Stable	3 (8.8)
Progressive	0 (0)
Pathologic TN stage	
Downstaging	29 (85.3)
Stable	5 (14.7)
Progressive	0 (0)

^a^Median (range)

^b^Two patients (T4bN2M0 and T4aN2M0) did not receive surgical resection due to unresectable tumor despite chemoradiotherapy

*ypT* postoperative pathologic tumor stage, *ypN* postoperative pathologic nodal stage, *CRM* circuferential resection margin

Table 4 summarizes the pathologic evaluation of primary tumor after neoadjuvant CCRT compared with that of the initial clinical stage. Of the 23 patients with clinical T4 tumors, five (21.7%) achieved ypT0 after intensified neoadjuvant CCRT, and clinical T4 tumors in eight patients (34.8%) were downstaged to ypT0-2.

**Table 4:** Comparison of clinical staging to pathologic T and N staging (*N* = 34)^a^

Clinical staging	Pathologic T staging						ypN negative	ypN positive	Total
	ypT0	ypT1	ypT2	ypT3	ypT4a	ypT4b			
cT3	4 (11.8)		2 (5.9)	7 (20.6)			-	-	13 (38.2)
cT4a	4 (11.8)		1 (2.9)	6 (17.6)	1 (3.0)		-	-	12 (35.3)
cT4b	1 (3.0)		2 (5.9)	4 (11.8)		2 (5.9)	-	-	9 (26.5)
cN negative	-	-	-	-	-	-	1 (3.0)	0	1 (3.0)
cN positive	-	-	-	-	-	-	27 (79.4)	6 (17.6)	33 (97.0)
Total	9 (26.5)	0	5 (14.7)	17 (50)	1 (3.0)	2 (5.9)	28 (82.4)	6 (17.6)	34 (100)

*c* clinical (in this case evaluated by imaging), *ypT* pathologic T-stage posttreatment, *ypN* pathologic N-stage posttreatment

^a^Two patients (T4bN2M0 and T4aN2M0) did not receive surgical resection due to unresectable tumor despite chemoradiotherapy

### Postoperative complications

Surgical mortality was not observed within 30 days after surgery. Of the 34 patients, five (14.7%) experienced postoperative complications requiring medical or surgical interventions. One patient developed a wound abscess on a previous drainage site, which resulted in a postoperative enterocutaneous fistula at 3 weeks, and this healed following antibiotic treatment. Another patient developed an intra-abdominal infection because of anastomotic leakage at 1 month after right hemicolectomy and temporary ileostomy closure; this patient finally recovered after undergoing another ileostomy along with antibiotic treatment. Two patients required surgical interventions because of adhesion ileus; one patient underwent enterolysis 1 month after left hemicolectomy and the other required segmental resection of small bowel at 32 months after extended left hemicolectomy. One patient developed a postoperative chronic rectovaginal fistula at 7 months and died of tumor progression.

### Failure patterns and survival data

**Fig. 1:**
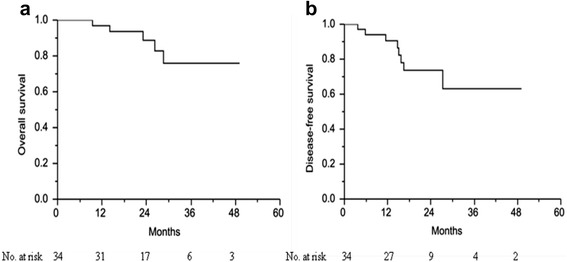
Overall and disease-free survival rates. **a** Overall survival rate and (**b**) disease-free survival rate in patients with locally advanced colon cancer receiving neoadjuvant chemoradiation and radical resection

Median follow-up period was 23.5 months (5.0–49.1 months). The 2-year estimated OS and DFS rates were 88.7 and 73.6%, respectively (Fig. 1). Of the 34 patients who completed neoadjuvant CCRT and radical resection, four (11.8%) experienced local recurrence and five (14.7%) developed distant metastasis. The most common first site of distant failure was lung metastasis (*n* = 2), followed by liver metastasis (*n* = 1), retroperitoneal lymph node metastasis (*n* = 1), and adrenal gland metastasis (*n* = 1). Univariate analysis of the correlation between local recurrence and clinicopathologic features, we found that positive surgical margins (*p* = 0.003), unfavorable tumor regression (TRG 3–4 versus TRG 0–1; *p* = 0.022), and ypT4 disease (*p* = 0.055) were associated with local recurrence.

## Discussion

According to our review of relevant literature, the present clinical study used the largest sample for investigating the role of combined neoadjuvant oxaliplatin-based chemoradiotherapy in the treatment of primary LACC [20]. Our data demonstrated that the intensified multimodality approach resulted in an excellent pCR rate of 26.4% with an acceptable toxicity profile. However, this multimodal therapy should be used cautiously in clinical practice because of the limited sample size and short follow-up period of the present study. Our results, nevertheless, suggest that intensified neoadjuvant CCRT should be considered as a treatment option for patients with LACC, particularly those with potentially threatened surgical resection margins.

Evidence has indicated that nodal involvement is a major predictor of oncologic outcomes in patients with colorectal cancer [21, 22]. In our study, 82.4% of the patients did not develop nodal diseases after neoadjuvant CCRT. Furthermore, in a surgical series, pathologically positive nodal involvement was observed in approximately 69% patients with LACC [23]. The high pN0 rate in our study may be attributable to the marked influence of the neoadjuvant CCRT. For locally advanced rectal cancer, the neoadjuvant CCRT has been associated with nodal downstaging and a decrease in the pathologic lymph node harvest [16, 24]. Our findings reveal a similar effect of the neoadjuvant CCRT on eradication of lymph node metastasis in LACC. However, OS and DFS rates did not differ significantly between the patients with and without nodal metastasis.

Evidence has demonstrated that patients with locally advanced rectal cancer who achieved a pCR to neoadjuvant CCRT exhibited excellent tumor control and survival rates [25]. Moreover, numerous studies have added oxaliplatin into neoadjuvant fluorouracil-base treatment to enhance pCR rates. Of the six large phase III trials (ACCORD 12/0405-Prodige 2, STAR-01, National Surgical Adjuvant Breast and Bowel Project R04, CAO/ARO/AIO-04, and PETTAC6), only CAO/ARO/AIO-04 demonstrated that the addition of oxaliplatin improved DFS and a pCR rate [26–30]. Garcia-Aguilar et al. reported that extending FOLFOX chemotherapy after neoadjuvant CCRT and before surgery in locally advanced rectal cancer resulted in a pCR arte of 38% [31]. The FOWARC trial demonstrated that FOLFOX chemotherapy administered concurrently with and following radiotherapy resulted in a higher pCR rate than fluorouracil-based CCRT or perioperative FOLFOX alone [8]. Our prior study also showed similar results to FOWARC trial [16]. However, the correlation between a pCR and clinical outcome in colon cancer has been rarely reported. To date, several studies have investigated the role of neoadjuvant chemotherapy in patients with LACC; however, the benefits of combined radiotherapy and chemotherapy for primary LACC have been rarely evaluated. Only a Canadian group reported that of the total 33 patients with LACC that were treated with neoadjuvant concurrent 5-FU and radiotherapy, only one patient (3%) achieved a pCR [14]. The FOxTROT trial assessed the role of neoadjuvant chemotherapy in LACC management by evaluating the efficacy and safety of the preoperative FOLFOX-based chemotherapy in a randomized controlled manner; two patients (2%) of the neoadjuvant group reported pCRs [32]. Furthermore, a phase II trial demonstrated that three (4.2%) of the 71 operated patients achieved a pCR after three cycles of neoadjuvant XELOX [capecitabine (2000 mg/m^2^) orally administered on days 1–14 (q3w) and oxaliplatin (130 mg/m^2^) intravenously infused on day 1 (q3w)] [33]. Arredondo et al. investigated 65 patients with LACC treated with either neoadjuvant XELOX- or FOLFOX-based chemotherapy; three patients (4.6%) had a pCR [34]. In the present study, patients with LACC were administered with neoadjuvant FOLFOX-based CCRT that resulted in a pCR rate of 26.4%, which was higher than that reported in other neoadjuvant studies (2–4.6%) [14, 33, 34].

The prognosis in patients with LACC remains discouraging even with the current standard treatment of radical resection followed by adjuvant chemotherapy. Danielle et al. analyzed the Surveillance, Epidemiology, and End Results Program database (1988–2008) and reported that the 5-year OS rates of patients with 7th AJCC stages IIC, IIIB, and IIIC were 54.6%, 59.0, 47.9%, respectively [35]. Therefore, treatment strategies should be modified to improve the oncologic outcomes in patients with LACC. Neoadjuvant CCRT is the standard of care in patients with locally advanced rectal cancer [6]. Although several studies have reported promising results for the neoadjuvant chemotherapy, we aimed to use an intensified multimodality approach of combined chemotherapy and radiotherapy in patients with LACC, particularly in those with clinical T4 tumors. Arredondo et al. investigated the efficacy and safety of neoadjuvant chemotherapy and reported that only 12 patients (18.5%) had T4 tumors [34]. Jakobsen et al. conducted a phase II trial in patients with LACC treated with neoadjuvant chemotherapy and reported that 11 (15%) patients developed T4 tumors [33]. Furthermore, the phase III FOxTROT trial reported on 30 patients (30%) with T4 tumors [32]. In our study, the proportion of patients with a T4 tumor was 63.9% (23 of the 36 patients), which is higher than that reported in other studies. The prognosis of a T4 colon cancer remains the worst, and T4 tumors have been closely associated with an involved resection margin [35, 36]. Therefore, we aimed to maximally treat LACC with a combination of radiotherapy and chemotherapy and obtained a R0 resection rate of 91.2%. Of the 23 patients with cT4 tumors in the present study, two (8.7%) did not undergo surgery because of a poor response to neoadjuvant CCRT, and 18 (85.7%) of the 21 patients received radical resection of cT4 tumors obtained R0 resections in our study.

Several studies have investigated the efficacy and safety of neoadjuvant chemotherapy for LACC [33, 37, 38]. The inclusion criteria for neoadjuvant chemotherapy was T4 or T3 with extramural extension >5 mm. By contrast, the eligibility for neoadjuvant CCRT was the tumor extensively involved other organs/structures on imaging studies or the tumor considered to be unresectable after exploratory laparotomy. Qiu et al. delivered neoadjuvant CCRT for 21 patients with unresectable locally advanced sigmoid colon cancer [20]. Of the 19 patients with clinical T4 diseases, seven patients achieved a pCR after CCRT. The results were similar to our study, in which five patients achieved a pCR after neoadjuvant CCRT among the 23 patients with clinical T4 tumors. For tolerability, both neoadjuvant chemotherapy and neoadjuvant CCRT were well-tolerable in patients with LACC, but toxicity was generally higher in neoadjuvant CCRT [14, 20, 32, 33]. Therefore, clinical T4 tumors, especially unresectable mass or tumors extensively involved adjacent structures, are potential indications for neoadjuvant CCRT. In addition, patients with a locally extensive T3 tumor or patients unable to tolerate CCRT may be candidates for neoadjuvant chemotherapy. However, the indications for neoadjuvant CCRT or chemotherapy need to be validated in more prospective randomized studies.

In en bloc multivisceral resections for primary locally advanced colorectal cancer, R0 resection rates ranged from 40 to 93%, with high postoperative complications (11%–40%) and a nonnegligible mortality up to 9% [36, 39, 40]. Cukier et al. analyzed the oncologic outcomes of neoadjuvant CCRT followed by multivisceral resection for primary LACC and reported a R0 resection rate of 100%, with a postoperative complication rate of 36% and zero surgical mortality [14]. Qiu et al. found that neoadjuvant CCRT can effectively reduce the peripheral tumor infiltration and thereby decrease the necessity for multivisceral resection. Therefore, neoadjuvant CCRT may decrease postoperative complications caused by multivisceral resection [20]. In the present study, the R0 resection rate was 91.2% with a postoperative complication rate of 14.7% and without surgical mortality, despite the intensified neoadjuvant CCRT. Our results suggested that neoadjuvant CCRT followed by radical resection does not increase a postoperative complication rate compared to resection alone (11%–40%).

However, the eligibility criterion of patients with LACC suitable for a neoadjuvant CCRT remains debatable. Moreover, the risk of overtreatment must be minimized because of occasional discrepancies between the clinical and pathologic stages. CT images have been primarily examined to select patients eligible to receive neoadjuvant therapy for colon cancer, and the accuracy of CT staging has improved. Numerous studies have demonstrated that CT can identify T3 tumors with extramural extension or T4 tumors, which are candidates for neoadjuvant therapy [28, 34, 37]. In contrast to tumor depth prediction, accurate nodal status prediction through CT remains difficult. Therefore, we assessed the CT images and T stage to guide neoadjuvant CCRT.

Cukier et al. reported that 9% of the patients with LACC experienced grade 3 or higher grades of adverse events during 5-FU-based CCRT [14]. In the present study, we used oxaliplatin and 5-FU in the neoadjuvant setting to maximize the effect of CCRT on LACC, and 19.4% of patients experienced grade 3 adverse events during neoadjuvant CCRT. Of all the patients, only one patient did not complete the prescribed radiation course, although FOLFOX-based CCRT had a relatively higher toxicity rate than that in 5-FU-based CCRT. However, the incidence of grade 3 or 4 adverse events reported in the present study were considerably lower than those reported in our previous studies or in other studies investigating the influence of FOLFOX-based CCRT in patients with rectal cancer, which ranged from 24% to 40% [16, 28, 37]. In our study, 88.9% of the patients received intensity-modulated radiotherapy with either VMAT or tomotherapy, which might partly contribute to the improved toxicity profiles because of normal organ sparing [41].

The present study has some limitations. First, because of the relatively small sample and short follow-up period, long-term efficacy and adverse events could not be addressed. Second, this was a retrospective study; therefore, the sampling may have been affected by selection bias. Third, the postoperative chemotherapy regimens were varied in our study, which might contribute to disease control and survival.

## Conclusions

Our results demonstrate that neoadjuvant chemoradiotherapy is feasible and safe. A high pCR and R0 resection rate with an acceptable toxicity profile suggests that multimodality therapy is a treatment option for patients with LACC. Additional prospective randomized studies are warranted to validate our results.

## Abbreviations

5-FU: 5-fluorouracil
AJCC: American Joint Committee on Cancer
CCRT: Concurrent chemoradiotherapy
CEA: Carcinoembryonic antigen
CRM: Circumferential resection margin
CT: Computed tomography
CTCAE: Common Terminology Criteria for Adverse Events
CTV: Clinical target volume
DFS: Disease-free survival
FOLFIRI: folinic acid, 5-fluorouracil, and irinotecan
FOLFOX: 5-fluorouracil, leucovorin, and oxaliplatin
GTV: Gross tumor volume
LACC: Locally advanced colon cancer
N: Nodal
OAR: Organs at risk
OS: Overall survival
pCR: Pathologic complete response
R0: Margin negative resection
T: Tumor
TRG: Tumor regression grade
UFUR: Tegafur and uracil
VMAT: Volumetric-modulated arc therapy
